# Diaqua­bis[5-(2-pyrid­yl)tetra­zolato-κ^2^ *N* ^1^,*N* ^5^]iron(II)

**DOI:** 10.1107/S1600536809007892

**Published:** 2009-03-11

**Authors:** Min Hu, Song-Tao Ma, Liang-Qi Guo, Shao-Ming Fang

**Affiliations:** a Zhengzhou University of Light Industry, Henan Provincial Key Laboratory of Surface & Interface Science, Henan, Zhengzhou 450002, People’s Republic of China

## Abstract

The title complex, [Fe(C_6_H_4_N_5_)_2_(H_2_O)_2_], was synthesized by the reaction of ferrous sulfate with 5-(2-pyrid­yl)-2*H*-tetra­zole (H*L*). The Fe^II^ atom, located on a crystallographic center of inversion, is coordinated by four N-atom donors from two planar *trans*-related deprotonated *L* ligands and two O atoms from two axial water mol­ecules in a distorted octa­hedral geometry. The Fe^II^ mononuclear units are further connected by inter­molecular O—H⋯N and C—H⋯O hydrogen-bonding inter­actions, forming a three-dimensional framework.

## Related literature

For hydrogen bonds, see: Desiraju & Steiner (1999 ▶); Kitagawa & Uemura (2005 ▶); For general background, see: Rizk *et al.* (2005 ▶); Robin & Fromm (2006 ▶); For structurally related complexes with tetra­zole ligands, see: Mo *et al.* (2004 ▶); Song *et al.* (2008 ▶); Tao *et al.* (2008 ▶); Wang *et al.* (2003 ▶); Wen (2008 ▶); Wu *et al.* (2007 ▶).
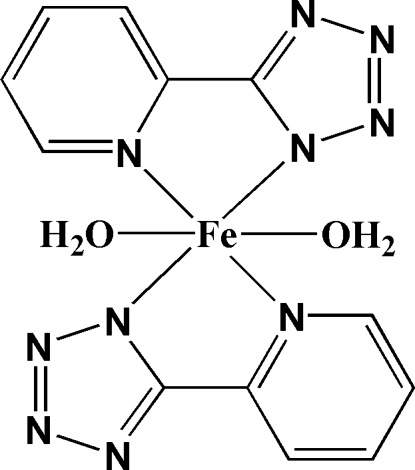


## Experimental

### 

#### Crystal data

[Fe(C_6_H_4_N_5_)_2_(H_2_O)_2_]
*M* *_r_* = 384.17
Monoclinic, 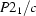

*a* = 8.114 (2) Å
*b* = 12.924 (3) Å
*c* = 7.360 (2) Å
β = 96.021 (3)°
*V* = 767.5 (3) Å^3^
*Z* = 2
Mo *K*α radiation
μ = 1.02 mm^−1^
*T* = 293 K
0.29 × 0.14 × 0.11 mm

#### Data collection

Bruker SMART CCD area-detector diffractometer
Absorption correction: multi-scan (*SADABS*; Sheldrick, 1996 ▶) *T*_min_ = 0.757, *T*_max_ = 0.897
4287 measured reflections
1356 independent reflections
1204 reflections with *I* > 2σ(*I*)
*R*_int_ = 0.017

#### Refinement

*R*[*F* ^2^ > 2σ(*F* ^2^)] = 0.023
*wR*(*F* ^2^) = 0.057
*S* = 1.10
1356 reflections
115 parameters
H-atom parameters constrained
Δρ_max_ = 0.23 e Å^−3^
Δρ_min_ = −0.24 e Å^−3^

### 

Data collection: *SMART* (Bruker, 1998 ▶); cell refinement: *SAINT* (Bruker, 1998 ▶); data reduction: *SAINT*; program(s) used to solve structure: *SHELXS97* (Sheldrick, 2008 ▶); program(s) used to refine structure: *SHELXL97* (Sheldrick, 2008 ▶); molecular graphics: *SHELXTL* (Sheldrick, 2008 ▶); software used to prepare material for publication: *SHELXTL* and *PLATON* (Spek, 2009 ▶).

## Supplementary Material

Crystal structure: contains datablocks I, global. DOI: 10.1107/S1600536809007892/im2104sup1.cif

Structure factors: contains datablocks I. DOI: 10.1107/S1600536809007892/im2104Isup2.hkl

Additional supplementary materials:  crystallographic information; 3D view; checkCIF report

## Figures and Tables

**Table 1:** Hydrogen-bond geometry (Å, °)

*D*—H⋯*A*	*D*—H	H⋯*A*	*D*⋯*A*	*D*—H⋯*A*
O1—H11⋯N5^i^	0.85	1.91	2.764 (2)	177
O1—H12⋯N4^ii^	0.85	2.00	2.823 (2)	162
C2—H2⋯O1^iii^	0.93	2.56	3.362 (3)	145

Symmetry codes: (i) 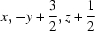; (ii) 
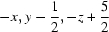
; (iii) 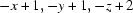.

**Fig. 1.**
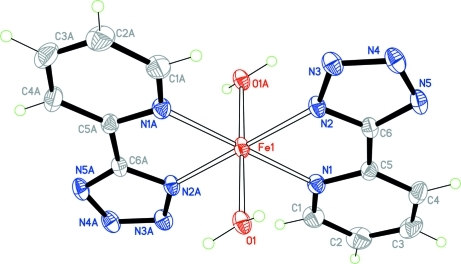
The molecular structure of the title complex. Displacement ellipsoids are drawn at the 30% probability level. Atoms labelled with the suffix A are generated by the symmetry operation (–x, –y + 1,–z + 2).

**Fig. 2.**
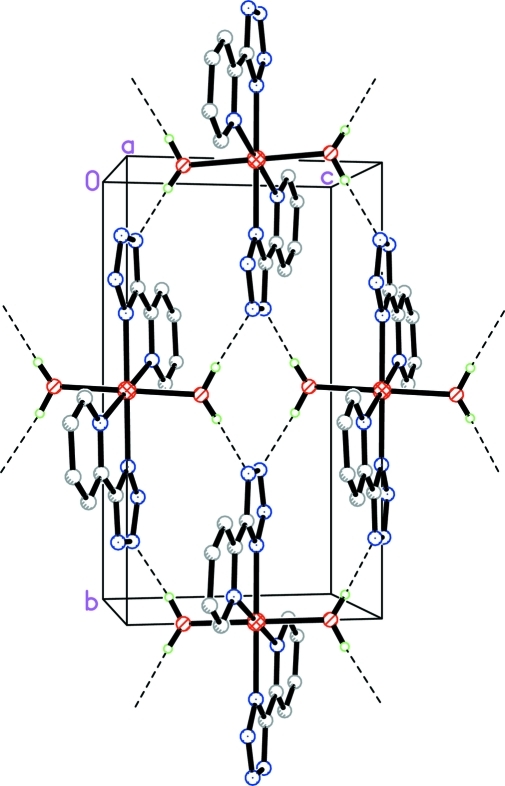
Two dimensional network, parallel to the (100) plane, formed by the intermolecular O—H···N (fine dashed lines) interactions. For clarity, only H atoms involved in the interactions are shown.

## supplementary crystallographic information

### Comment

Currently, there is considerable interest in self-assembly and construction of supramolecular complexes featuring fascinating architectures realized by noncovalent interactions such as coordination bonds, hydrogen bonds and other weak intermolecular interactions (Rizk *et al.*, 2005; Robin & Fromm, 2006). Hydrogen bonds, combining directionality, strength and selectivity, have been noted as the most versatile organizing force to assemble supramolecular structures (Kitagawa & Uemura, 2005). Due to their ability of providing multi-coordination sites as well as hydrogen bonding acceptors, tetrazole and its derivatives are receiving much attention in coordination and supramolecular chemistry (Mo *et al.*, 2004; Song *et al.*, 2008; Tao *et al.*, 2008; Wang *et al.*, 2003; Wen, 2008; Wu *et al.*, 2007). Herein we report the crystal structure of an iron(II) complex of 2-pyridyl-tetrazole *HL*, [Fe(*L*)_2_(H_2_O)_2_].

In the title complex, the Fe^II^ atom is located on a crystallographic center of inversion. It is  six-coordinated by two O atoms from water molecules and four N-atom donors from two deprotonated *N,N'*-chelating *L* ligands binding *via* the pyridyl nitrogen and the tetrazole nitrogen in 1-position, in a *transoid* pseudo-octahedral geometry (Fig. 1).

Each Fe^II^ mononuclear unit exhibits both proton donors (water molecules) and acceptors (uncoordinated N atoms on the tetrazole rings) and can therefore act as a good building unit for hydrogen bonded networks. As shown in Fig. 2 the O—H···N hydrogen bonds (Table 1) between tetrazole rings and coordinated water molecules link neighboring mononuclear [Fe(*L*)_2_(H_2_O)_2_] units resulting in an infinite hydrogen bonded layer running parallel to the crystallographic (100) plane. Furthermore, the crystal structure of (I) also contains intermolecular C—H···O (Table 1) hydrogen-bonding interactions (Desiraju & Steiner, 1999), between the *L* ligands and the coordinated water molecules that interlink the twodimensional layers to form a three dimensional supramolecular framework.

### Experimental

A solution of H*L* (0.05 mmol) in CH_3_OH (10 ml) in the presence of excess 2,6-dimethylpyridine (*ca* 0.05 ml for adjusting the pH value of the reaction system to basic conditions) was carefully layered on top of an aqueous solution (15 ml) of FeSO_4_ (0.1 mmol) in a test tube. Yellow single crystals suitable for X-ray analysis appeared at the tube wall after *ca* one month at room temperature (yield ~30% based on H*L*). Elemental analysis calculated for (C_12_H_12_FeN_10_O_2_): H 3.15, C 37.52, N 36.46%; found: H 3.08, C 37.37, N 36.68%.

### Refinement

H atoms of the water molecules were located from the difference Fourier map and were allowed to ride on the O atom, with *U*_iso_(H) = 1.2 *U*_eq_(O). The remaining H atoms were included in calculated positions and treated in the subsequent refinement as riding atoms, with C—H = 0.93 (aromatic), and *U*_iso_(H) = 1.2 *U*_eq_(C).

### Crystal data

**Table:**

[Fe(C_6_H_4_N_5_)_2_(H_2_O)_2_]	*F*(000) = 392
*M**_r_* = 384.17	*D*_x_ = 1.662 Mg m^−^^3^
Monoclinic, *P*2_1_/*c*	Mo *K*α radiation, λ = 0.71073 Å
Hall symbol: -P 2ybc	Cell parameters from 2211 reflections
*a* = 8.114 (2) Å	θ = 3.0–28.3°
*b* = 12.924 (3) Å	µ = 1.01 mm^−^^1^
*c* = 7.360 (2) Å	*T* = 293 K
β = 96.021 (3)°	Block, yellow
*V* = 767.5 (3) Å^3^	0.29 × 0.14 × 0.11 mm
*Z* = 2	

### Data collection

**Table:**

Bruker SMART CCD area-detector diffractometer	1356 independent reflections
Radiation source: fine-focus sealed tube	1204 reflections with *I* > 2σ(*I*)
graphite	*R*_int_ = 0.017
φ and ω scans	θ_max_ = 25.0°, θ_min_ = 3.0°
Absorption correction: multi-scan (*SADABS*; Sheldrick, 1996)	*h* = −9→8
*T*_min_ = 0.757, *T*_max_ = 0.897	*k* = −15→15
4287 measured reflections	*l* = −8→8

### Refinement

**Table:**

Refinement on *F*^2^	Primary atom site location: structure-invariant direct methods
Least-squares matrix: full	Secondary atom site location: difference Fourier map
*R*[*F*^2^ > 2σ(*F*^2^)] = 0.023	Hydrogen site location: inferred from neighbouring sites
*wR*(*F*^2^) = 0.057	H-atom parameters constrained
*S* = 1.10	*w* = 1/[σ^2^(*F*_o_^2^) + (0.0207*P*)^2^ + 0.3154*P*] where *P* = (*F*_o_^2^ + 2*F*_c_^2^)/3
1356 reflections	(Δ/σ)_max_ < 0.001
115 parameters	Δρ_max_ = 0.23 e Å^−^^3^
0 restraints	Δρ_min_ = −0.24 e Å^−^^3^

### Special details

**Table:**

Geometry. All e.s.d.'s (except the e.s.d. in the dihedral angle between two l.s. planes) are estimated using the full covariance matrix. The cell e.s.d.'s are taken into account individually in the estimation of e.s.d.'s in distances, angles and torsion angles; correlations between e.s.d.'s in cell parameters are only used when they are defined by crystal symmetry. An approximate (isotropic) treatment of cell e.s.d.'s is used for estimating e.s.d.'s involving l.s. planes.
Refinement. Refinement of *F*^2^ against ALL reflections. The weighted *R*-factor *wR* and goodness of fit *S* are based on *F*^2^, conventional *R*-factors *R* are based on *F*, with *F* set to zero for negative *F*^2^. The threshold expression of *F*^2^ > σ(*F*^2^) is used only for calculating *R*-factors(gt) *etc*. and is not relevant to the choice of reflections for refinement. *R*-factors based on *F*^2^ are statistically about twice as large as those based on *F*, and *R*- factors based on ALL data will be even larger.

### Fractional atomic coordinates and isotropic or equivalent isotropic displacement parameters (Å^2^)

**Table:**

	*x*	*y*	*z*	*U*_iso_*/*U*_eq_	
Fe1	0.0000	0.5000	1.0000	0.02908 (12)	
C1	0.3360 (2)	0.51552 (16)	0.8123 (3)	0.0456 (5)	
H1	0.3306	0.4437	0.8065	0.055*	
C2	0.4708 (3)	0.56508 (19)	0.7511 (3)	0.0565 (6)	
H2	0.5543	0.5270	0.7050	0.068*	
C3	0.4800 (3)	0.67167 (19)	0.7593 (3)	0.0539 (6)	
H3	0.5708	0.7063	0.7213	0.065*	
C4	0.3524 (2)	0.72590 (16)	0.8246 (3)	0.0440 (5)	
H4	0.3550	0.7978	0.8296	0.053*	
C5	0.2204 (2)	0.67171 (13)	0.8826 (2)	0.0318 (4)	
C6	0.0757 (2)	0.72097 (12)	0.9502 (2)	0.0304 (4)	
N1	0.21327 (18)	0.56715 (11)	0.87949 (19)	0.0332 (3)	
N2	−0.04622 (17)	0.66384 (10)	1.00861 (18)	0.0308 (3)	
N3	−0.15883 (19)	0.73201 (11)	1.0590 (2)	0.0367 (4)	
N4	−0.1046 (2)	0.82582 (11)	1.0311 (2)	0.0396 (4)	
N5	0.0442 (2)	0.82160 (11)	0.9625 (2)	0.0375 (4)	
O1	0.13384 (15)	0.50897 (8)	1.26584 (16)	0.0340 (3)	
H11	0.1091	0.5606	1.3292	0.041*	
H12	0.1461	0.4509	1.3199	0.041*	

### Atomic displacement parameters (Å^2^)

**Table:**

	*U*^11^	*U*^22^	*U*^33^	*U*^12^	*U*^13^	*U*^23^
Fe1	0.0345 (2)	0.01947 (19)	0.0352 (2)	0.00073 (14)	0.01281 (15)	0.00110 (14)
C1	0.0410 (11)	0.0464 (12)	0.0517 (12)	0.0074 (9)	0.0153 (9)	0.0027 (9)
C2	0.0407 (13)	0.0745 (16)	0.0573 (13)	0.0099 (11)	0.0195 (10)	0.0056 (12)
C3	0.0357 (11)	0.0773 (17)	0.0497 (12)	−0.0136 (11)	0.0102 (9)	0.0086 (11)
C4	0.0444 (12)	0.0457 (11)	0.0423 (10)	−0.0154 (9)	0.0069 (9)	0.0037 (9)
C5	0.0352 (9)	0.0332 (10)	0.0268 (8)	−0.0052 (7)	0.0020 (7)	0.0027 (7)
C6	0.0409 (10)	0.0249 (9)	0.0253 (8)	−0.0037 (7)	0.0028 (7)	0.0007 (7)
N1	0.0343 (8)	0.0313 (8)	0.0355 (8)	0.0010 (6)	0.0099 (6)	0.0028 (6)
N2	0.0378 (8)	0.0215 (7)	0.0346 (8)	0.0018 (6)	0.0103 (6)	−0.0002 (6)
N3	0.0449 (9)	0.0274 (8)	0.0390 (8)	0.0062 (7)	0.0091 (7)	−0.0022 (6)
N4	0.0564 (10)	0.0255 (8)	0.0371 (8)	0.0046 (7)	0.0064 (7)	−0.0017 (6)
N5	0.0543 (10)	0.0223 (7)	0.0358 (8)	−0.0032 (7)	0.0040 (7)	0.0015 (6)
O1	0.0434 (7)	0.0226 (6)	0.0373 (7)	0.0035 (5)	0.0105 (5)	0.0006 (5)

### Geometric parameters (Å, °)

**Table:**

Fe1—O1^i^	2.1389 (13)	C3—H3	0.9300
Fe1—O1	2.1389 (13)	C4—C5	1.384 (2)
Fe1—N2	2.1526 (14)	C4—H4	0.9300
Fe1—N2^i^	2.1526 (14)	C5—N1	1.353 (2)
Fe1—N1^i^	2.2037 (14)	C5—C6	1.468 (2)
Fe1—N1	2.2037 (14)	C6—N5	1.330 (2)
C1—N1	1.336 (2)	C6—N2	1.341 (2)
C1—C2	1.383 (3)	N2—N3	1.3489 (19)
C1—H1	0.9300	N3—N4	1.313 (2)
C2—C3	1.380 (3)	N4—N5	1.358 (2)
C2—H2	0.9300	O1—H11	0.8501
C3—C4	1.378 (3)	O1—H12	0.8500
			
O1^i^—Fe1—O1	180.0	C2—C3—H3	120.5
O1^i^—Fe1—N2	90.40 (5)	C3—C4—C5	118.96 (19)
O1—Fe1—N2	89.60 (5)	C3—C4—H4	120.5
O1^i^—Fe1—N2^i^	89.60 (5)	C5—C4—H4	120.5
O1—Fe1—N2^i^	90.40 (5)	N1—C5—C4	122.25 (17)
N2—Fe1—N2^i^	180.0	N1—C5—C6	113.85 (14)
O1^i^—Fe1—N1^i^	90.12 (5)	C4—C5—C6	123.89 (16)
O1—Fe1—N1^i^	89.88 (5)	N5—C6—N2	111.28 (15)
N2—Fe1—N1^i^	103.24 (5)	N5—C6—C5	127.83 (15)
N2^i^—Fe1—N1^i^	76.76 (5)	N2—C6—C5	120.89 (14)
O1^i^—Fe1—N1	89.89 (5)	C1—N1—C5	118.22 (16)
O1—Fe1—N1	90.11 (5)	C1—N1—Fe1	126.83 (13)
N2—Fe1—N1	76.76 (5)	C5—N1—Fe1	114.89 (11)
N2^i^—Fe1—N1	103.24 (5)	C6—N2—N3	105.81 (13)
N1^i^—Fe1—N1	180.000 (1)	C6—N2—Fe1	113.29 (11)
N1—C1—C2	122.33 (19)	N3—N2—Fe1	140.87 (11)
N1—C1—H1	118.8	N4—N3—N2	108.19 (14)
C2—C1—H1	118.8	N3—N4—N5	110.28 (13)
C3—C2—C1	119.3 (2)	C6—N5—N4	104.44 (14)
C3—C2—H2	120.4	Fe1—O1—H11	114.7
C1—C2—H2	120.4	Fe1—O1—H12	113.8
C4—C3—C2	118.92 (19)	H11—O1—H12	117.3
C4—C3—H3	120.5		
			
N1—C1—C2—C3	0.0 (3)	N2—Fe1—N1—C5	5.08 (12)
C1—C2—C3—C4	−1.4 (3)	N2^i^—Fe1—N1—C5	−174.92 (12)
C2—C3—C4—C5	1.0 (3)	N5—C6—N2—N3	0.17 (19)
C3—C4—C5—N1	0.8 (3)	C5—C6—N2—N3	−179.46 (14)
C3—C4—C5—C6	−178.37 (17)	N5—C6—N2—Fe1	−178.30 (11)
N1—C5—C6—N5	−177.20 (16)	C5—C6—N2—Fe1	2.07 (19)
C4—C5—C6—N5	2.1 (3)	O1^i^—Fe1—N2—C6	−93.43 (12)
N1—C5—C6—N2	2.4 (2)	O1—Fe1—N2—C6	86.57 (12)
C4—C5—C6—N2	−178.36 (16)	N1^i^—Fe1—N2—C6	176.36 (11)
C2—C1—N1—C5	1.8 (3)	N1—Fe1—N2—C6	−3.64 (11)
C2—C1—N1—Fe1	−175.34 (15)	O1^i^—Fe1—N2—N3	88.90 (18)
C4—C5—N1—C1	−2.2 (3)	O1—Fe1—N2—N3	−91.10 (18)
C6—C5—N1—C1	177.07 (16)	N1^i^—Fe1—N2—N3	−1.31 (18)
C4—C5—N1—Fe1	175.23 (13)	N1—Fe1—N2—N3	178.69 (18)
C6—C5—N1—Fe1	−5.48 (18)	C6—N2—N3—N4	−0.01 (18)
O1^i^—Fe1—N1—C1	−87.30 (16)	Fe1—N2—N3—N4	177.76 (13)
O1—Fe1—N1—C1	92.70 (16)	N2—N3—N4—N5	−0.15 (19)
N2—Fe1—N1—C1	−177.74 (17)	N2—C6—N5—N4	−0.26 (19)
N2^i^—Fe1—N1—C1	2.26 (17)	C5—C6—N5—N4	179.35 (16)
O1^i^—Fe1—N1—C5	95.51 (12)	N3—N4—N5—C6	0.25 (19)
O1—Fe1—N1—C5	−84.49 (12)		

Symmetry codes: (i) −*x*, −*y*+1, −*z*+2.

### Hydrogen-bond geometry (Å, °)

**Table:**

*D*—H···*A*	*D*—H	H···*A*	*D*···*A*	*D*—H···*A*
O1—H11···N5^ii^	0.85	1.91	2.764 (2)	177
O1—H12···N4^iii^	0.85	2.00	2.823 (2)	162
C2—H2···O1^iv^	0.93	2.56	3.362 (3)	145

Symmetry codes: (ii) *x*, −*y*+3/2, *z*+1/2; (iii) −*x*, *y*−1/2, −*z*+5/2; (iv) −*x*+1, −*y*+1, −*z*+2.
